# 

**Published:** 1920-10-16

**Authors:** 


### Important Gift to a Cottage Hospital.

THE Tredegar Medical-Aid Society has presented an elaborate *x*-ray apparatus, costing nearly £800, to the Tredegar Park cottage Hospital. When making the gift, Mr. George Davies, Chairman of the Society, explained that it was for the use of the whole community. It is stated, we understand, that only three larger installations exist in this country, all of which are in London hospitals.

### "Care of Children."

A NEW edition (the fourth) of "Care of Children," by Dr. Robert Blackham, C.B., D.S.O., will be issued shortly by The Scientific Press, Ltd., 28 and 29 Southampton Street, Strand, London, W.C. 2. The book has been thoroughly revised, much enlarged, and in part re-written. It has been largely used in connection with child-welfare centres at home and in India, and will be found a trustworthy guide to all concerned in the care of children.

### The College of Nursing

### Matrons' and Sisters' Appointments.

Falmouth Cottage Hospital.—Miss Nellie M. Roper has been appointed matron. She was trained at the South Devon and East Cornwall Hospital, and has served on the staff of the 1st Eastern General Hospital, Cambridge, and as sister at the West Cornwall Infirmary, Penzance, and the Royal Cornwall Sailors' Hospital, Falmouth.

Clackmannan County Accident Hospital.—Miss Jean Henry has been appointed matron. She was trained at the Western Infirmary, Glasgow, where she was later ward sister. She has served under the Territorial Force Nursing Service and as sister at Clackmannan County Accident Hospital.

Isolation Hospital, Rawmarsh.—Miss E. Ellis has been appointed matron. She was trained at the Monsall Hospital, Manchester, and has held the post of matron of the following hospitals: Isolation Hospital, Tewkesbury; Joint Hospital, Tredington, Tewkesbury; and the Isolation Hospital, Upton-on-Severn.

Sidlaw Sanatorium, Auchterhouse.—Miss Ellen Norris has been appointed matron. She was trained at Dundee Royal Infirmary, where she was subsequently sister of the male medical wards, sister of the soldiers' wards, and matron's office assistant. Before taking her general training at Dundee, Miss Norris was matron of the Macalpine Maternity Home, Manchester.

Camberwell Poor-Law Infirmary.—Miss M. E. Jones has been appointed matron. She was trained at the Infirmary, West Bromwich, and subsequently held the following posts: Ward sister, Camberwell Infirmary and Whipps Cross Infirmary; assistant matron, City of Westminster Infirmary; and assistant matron, Whipps Cross (West Ham) Hospital, which post she has held since 1916.

Severalls Mental Hospital, Colchester.—Miss E. M. Cuthbert has been appointed matron. She was trained at the Eye and Ear Infirmary, Liverpool, and Southport General Infirmary, and has since been ward, theatre, and out-patient sister at the Eye and Ear Infirmary, Liverpool, and out-patient sister at the Central London Ear and Throat Hospital, Gray's Inn Road, W.C., night superintendent at Montrose Royal Asylum, assistant matron at Edinburgh Royal Asylum, and matron and housekeeper at Sunderland Mental Hospital, Ryhope. She is a member of the College of Nursing, and holds the certificate granted for mental nursing.

Infectious Diseases Hospital, Blaydon-on-Tyne.—Miss H. Powell Evans, A.R.R.C., has been appointed matron. She was trained at the Manchester Royal Infirmary and after her fever at Belvidere Hospital, Glasgow, where she after-wards held the post of sister. She has done some private nursing. Miss Evans belongs to the Territorial Force Nursing Service, and has done military work in Oxford, Malta, and Salonika, and was Mentioned in Despatches. She is a member of the College of Nursing.

Victoria Cottage Hospital, Maryport.—Miss Mabel Scott has been appointed nurse-matron. She was trained at St. Bartholomew's Hospital and the Chelsea Hospital for Women, and has been sister at the Adelaide Hospital, Dublin.

